# A Method to Evaluate Orientation-Dependent Errors in the Center of Contrast Targets Used with Terrestrial Laser Scanners

**DOI:** 10.3390/s25020505

**Published:** 2025-01-16

**Authors:** Bala Muralikrishnan, Xinsu Lu, Mary Gregg, Meghan Shilling, Braden Czapla

**Affiliations:** 1 Sensor Science Division, National Institute of Standards and Technology, Gaithersburg, MD 20878, USA; xinsuelu@gmail.com (X.L.); katharine.shilling@nist.gov (M.S.); braden.czapla@nist.gov (B.C.); 2 Thomas Wootton High School, Rockville, MD 20850, USA; 3 Statistical Engineering Division, National Institute of Standards and Technology, Boulder, CO 80305, USA; mary.gregg@nist.gov

**Keywords:** center error, checkerboard, contrast target, forensics, sphere target, terrestrial laser scanner

## Abstract

Terrestrial laser scanners (TLS) are portable dimensional measurement instruments used to obtain 3D point clouds of objects in a scene. While TLSs do not require the use of cooperative targets, they are sometimes placed in a scene to fuse or compare data from different instruments or data from the same instrument but from different positions. A contrast target is an example of such a target; it consists of alternating black/white squares that can be printed using a laser printer. Because contrast targets are planar as opposed to three-dimensional (like a sphere), the center of the target might suffer from errors that depend on the orientation of the target with respect to the TLS. In this paper, we discuss a low-cost method to characterize such errors and present results obtained from a short-range TLS and a long-range TLS. Our method involves comparing the center of a contrast target against the center of spheres and, therefore, does not require the use of a reference instrument or calibrated objects. For the short-range TLS, systematic errors of up to 0.5 mm were observed in the target center as a function of the angle for the two distances (5 m and 10 m) and resolutions (30 points-per-degree (ppd) and 90 ppd) considered for this TLS. For the long-range TLS, systematic errors of about 0.3 mm to 0.8 mm were observed in the target center as a function of the angle for the two distances (5 m and 10 m) at low resolution (28 ppd). Errors of under 0.3 mm were observed in the target center as a function of the angle for the two distances at high resolution (109 ppd).

## 1. Introduction

Terrestrial laser scanners (TLSs) [1,2] measure the range, the azimuth angle, and the elevation angle of points on surfaces to produce a three-dimensional map of objects in a scene. They do not require cooperative targets (such as a spherically mounted retro-reflector (SMR) for a laser tracker). Instead, they measure passive reflectance from objects to estimate range. They are also able to report the intensity of the return signal along with a 3D coordinate for every measured point. TLSs are increasingly used in surveying and geodesy, manufacturing and assembly of large structures, reverse engineering, historical monument digitization and preservation, and forensic applications such as crime scene digitization and reconstruction.

**Figure 1:**
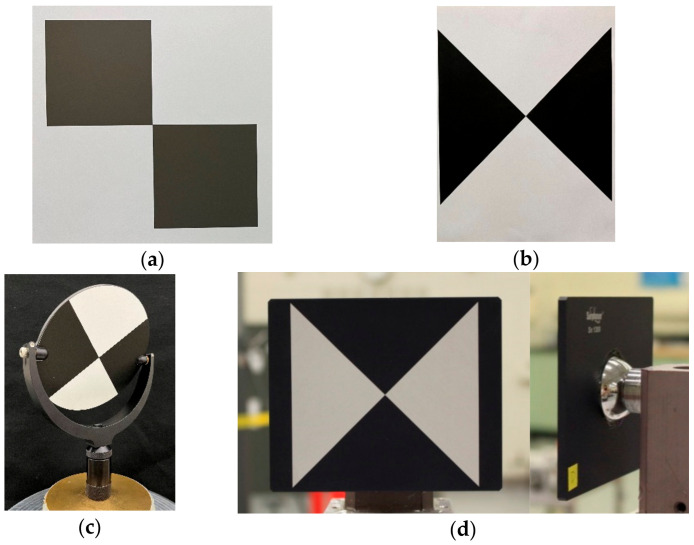
(**a**) Commercially procured contrast target with magnetic/adhesive backing, (**b**) contrast target printed on cardstock using a laser printer, (**c**) contrast target mounted on a two-axis gimbal, (**d**) contrast target with a partial 38.1 mm (1.5 inches) sphere on the back.

Although not required, targets are used in special cases such as for registering point clouds acquired from different TLS stations, calibrating TLSs, characterizing the performance of TLSs in accordance with published documentary standards such as the ASTM E3125-17 [3], fusing data acquired from different measurement technologies such as a TLS and a camera, etc. Spheres and signalized planar targets (such as those with circles, triangles, squares, and other shapes that can be reduced to a single point) are commonly used for these purposes as the point cloud from these targets can be reduced to a single point, the “center” of the target. Contrast targets considered in this paper are the simplest of these signalized targets, consisting of alternating black and white geometric features such as triangles or squares, see Figure 1a,b. These are also sometimes referred to as checkerboard, chess board, or black and white targets. They can be printed on a laser printer and are therefore inexpensive and easily accessible. The center of the target is typically determined using both geometric as well as intensity information from the point cloud using software provided by the manufacturer of the TLS.

While the three-dimensional geometry of a sphere is ideally suited for a TLS because a sphere appears the same from any view, there is still a cost associated with procuring spheres with acceptable form error (sphericity) and finish, especially if multiple targets are required to be placed in a scene. Contrast targets, on the other hand, are inexpensive and easily available. However, their planar geometry might result in errors in the center location that depend on the orientation of the target with respect to the TLS. These errors likely arise because of a combination of factors—the finite spot size of the TLS laser beam and its effect near the edges of surfaces (such as the transition region between the black and white surfaces) [4,5], the resolution of the TLS and the nature in which the data are acquired (in vertical columns), and the algorithm used to reduce that data to a single point, i.e., the target center.

A simple approach to studying the effect of target orientation on center coordinate error might involve using commercially available contrast targets such as shown in Figure 1c,d. The target in Figure 1c is mounted on a two-axis gimbal allowing the target orientation to be changed with respect to the TLS. Such targets are sometimes referred to as tip/tilt or paddle targets. The target in Figure 1d has a partial 38.1 mm (1.5 inch) sphere mounted on the back so that the center of the sphere is designed to be coincident with the center of the target on the front face. This allows the target to be mounted on 38.1 mm (1.5 inch) SMR nests; thus, the target center location can also be measured using a laser tracker and an SMR. Ideally, these targets should produce the same center even as their orientation is changed, thus potentially allowing for a simple technique to quantify the orientation-dependent error. However, the error introduced by the two-axis gimbal in case (c) and the error between the mechanical and optical center in case (d) can be on the order of several tenths of a millimeter [6], thus introducing a new error source into the mix. In addition, these targets can be expensive to procure, making it a challenge for many users.

In this context, we present a method to quantify the error in the contrast target center as the target orientation is changed with respect to the TLS. Our method does not require the use of a reference instrument or calibrated objects, instead, we rely on spheres as the reference against which we compare the center of the contrast target, for different orientations of the target with respect to the TLS. The motivation for this work is an ongoing standards effort within the Crime Scene Investigation and Reconstruction sub-committee of the Organization for Scientific Area Committees for Forensic Science (OSAC) [7] to develop a TLS test procedure for forensic practitioners to use in the field. The procedure being developed relies on the use of contrast targets, hence the need to better understand the errors introduced as a function of target orientation. The rest of the paper is organized as follows. We present a review of the literature in Section 2, a summary of our approach in Section 3, results in Section 4, and conclusions in Section 5.

## 2. Literature Review

There is considerable literature on the use of targets in conjunction with TLSs. The review presented here is limited to the use of contrast targets as that is the focus of this paper.

As we noted in the previous section, contrast targets are used to calibrate TLSs, i.e., evaluate the parameters of the error model of the TLS. See Muralikrishnan [8] for a review of the literature on this topic. Such calibration can be performed with or without the use of reference artifacts or reference instruments. Self-calibration is the process of evaluating the parameters of the error model of a TLS from measurements made on stationary targets from multiple positions of the TLS, without the need for any calibrated reference artifacts or reference instrument. Abbas et al. [9] describe the calibration of a TLS using contrast targets. They use photogrammetry to establish the distances between pairs of targets and compare the performance of the TLS before and after calibration. Wang et al. [10] present three approaches for self-calibrating a TLS using a network of contrast targets while Muralikrishnan et al. [11] present the underlying mathematics. Medic et al. [12] discuss the evaluation of the model parameters of a TLS using a network of contrast targets, but from only a single station of the TLS, thus offering a simpler approach to evaluating the model parameters. Shi et al. [13] explore the topic of whether the network must be calibrated using an instrument of higher accuracy such as a laser tracker (LT) or whether the TLS under study is itself suitable for network calibration. They provide guidance on when a self-calibrated network may be used for TLS calibration. Calibrating the parameters of a TLS using contrast targets and a total station as a reference instrument is discussed by Reshetyuk [14] and García-San-Miguel and Lerma [15]. The method presented by Reshetyuk reduces the correlations between the parameters but requires independent information to determine the TLS rigid body transformation parameters. Muralikrishnan et al. [16] use a laser tracker as a reference instrument to calibrate the parameters of a TLS.

Performance evaluation is the process of assessing the magnitude of errors in a TLS measurement and/or determining whether the TLS meets its own specifications. This is often performed using calibrated artifacts in a controlled environment, and typically, in accordance with published documentary standards. Staiger [17] and Kersten et al. [18] present early studies in this area where they use contrast targets and reference instruments to verify the distance performance of TLSs. Wunderlich and Wasmeier [19] present results from an extensive series of tests conducted on TLSs and contrast targets were used for some of them. Walser and Gordon [20] describe a test procedure used to evaluate TLSs that uses contrast targets, these tests were later incorporated into the ISO 17123-9 standard [21] and in the appendix of the ASTM E3125-17 standard [3]. Other studies that involve the use of contrast targets for performance evaluation can be found in [22,23,24,25,26,27].

Spheres and signalized planar targets are typically used for registration, i.e., fusing data from different stations of a TLS. See Cheng et al. [28] and Dong et al. [29] for a review of the literature on this topic. Akca [30] and Yi et al. [31] present an automated method to recognize contrast targets in scans so that the data from the different stations can be registered together. The effect of different target types and layouts on registration accuracy is discussed by Becerik-Gerber [32] et al. Their study includes contrast targets printed on paper as well as those mounted on two-axis gimbal such as shown in Figure 1c. Stenz et al. [33] address the general problem of fusing multi-sensor data with TLSs and the implications for accuracy and uncertainty.

Within the domain of forensics, Berezowski et al. [34] and Raneri [35] discuss the use of geomatic techniques and provide a summary of laser scanning used in forensics. The commercially available twin-target pole, calibrated by the National Institute of Standards and Technology (NIST) [36], can be used to verify the performance of TLS in the field by forensic practitioners. Liscio et al. [37] study the effectiveness of TLSs in forensics, especially on data registered using contrast targets. Targets can not only be used to fuse data from different stations of a single TLS but also to fuse data from different instruments, such as a TLS and a camera. Kwan et al. [38] discuss the fusing of camera and TLS data to analyze blood stains and identify trajectories. Berezowski et al. [39] discuss registering scans using contrast targets for the case of a human burial grave scene documentation. Dustin and Liscio [40] discuss the use of a TLS for crime scene documentation, in particular, they study the accuracy of a TLS in comparison to a total station and laser tracker, using a specially fabricated contrast target that accommodates an SMR in the middle.

Janβen et al. [41] provide a summary of methods used to estimate the center of a contrast target. They classify the techniques into three categories: averaging, edge-based, and correlation-based techniques. Ge and Wunderlich [42] and Rachakonda et al. [43,44] present edge-based techniques. Abmayr et al. [45] present a correlation-based technique. Rosa [46] presents a study of contrast target designs specifically to improve the accuracy in center detection. Fryskowska [47] presents an approach to improve target centers for the case of low-resolution and low-quality point clouds. Liang et al. [48] focus on the rapid detection of multiple targets in a scene.

## 3. Approach

**Figure 2:**
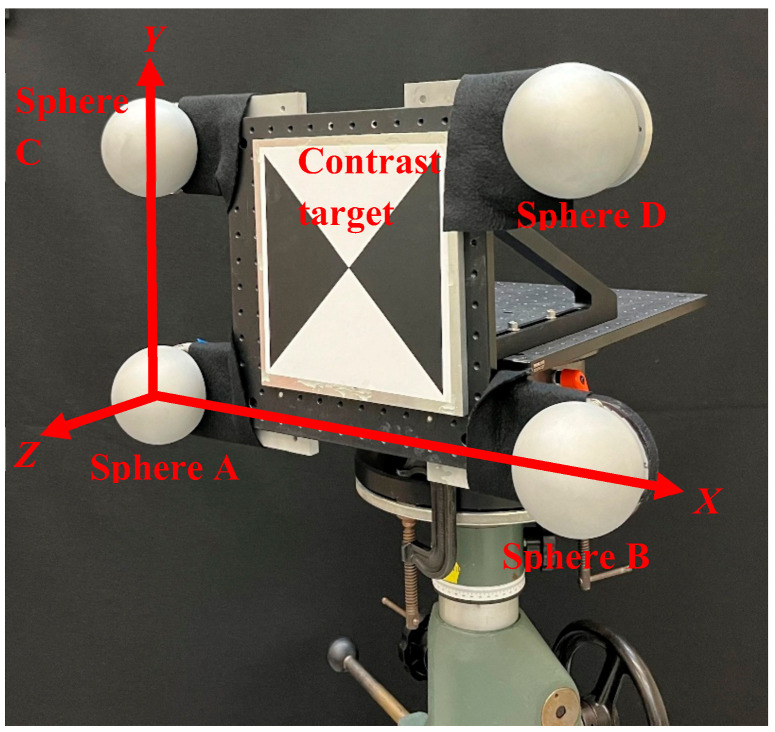
Artifact comprising four spheres and a contrast target to study errors as a function of orientation.

**Figure 3:**
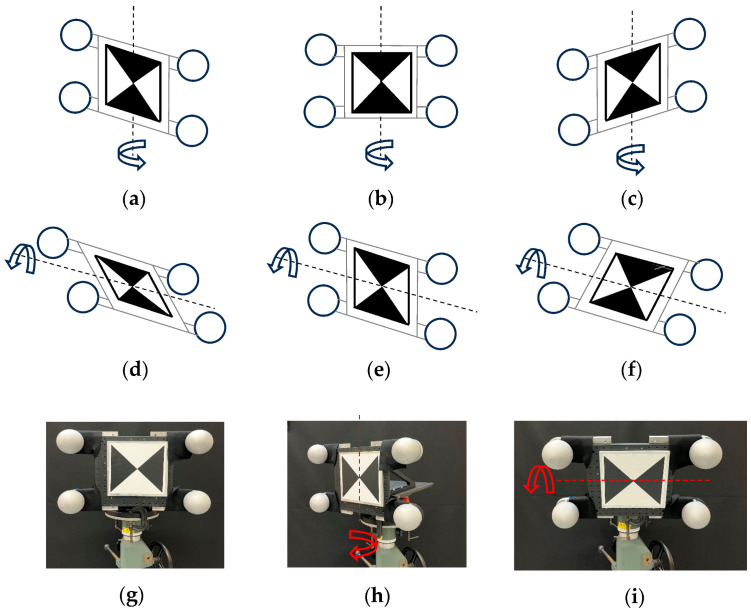
Different orientations of the artifact, (**a**–**c**) rotation about the vertical axis, i.e., yaw, (**d**–**f**) rotation about the horizontal axis, i.e., pitch. Photos of the artifact oriented so that (**g**) yaw = 0°, pitch = 0°, (**h**) yaw = 40°, pitch = 0°, (**i**) yaw = 0°, pitch = −40°. The TLS is located directly in front of the target in part (**g**) at a distance of either 5 m or 10 m.

We evaluate the orientation-dependent errors in a contrast target using the artifact shown in Figure 2. It is comprised of four spheres and a contrast target mounted on an aluminum breadboard. The spheres are 100 mm in diameter, made of aluminum with a dull gray finish, and sphericity better than 10 µm. A 250 mm × 250 mm contrast target is printed on cardstock using a laser printer and glued onto a flat aluminum plate, which is bolted to the breadboard. The artifact is mounted on two rotation stages to allow different orientations (rotations about X and Y), as shown in Figure 3, to be presented to the TLS. The grayscale intensity of the black portion of the contrast target was chosen so that both TLSs can acquire data even at large angles of incidence. We have not considered the effect of other intensities in this paper. The flatness of the target is limited by the flatness of the aluminum plate and the extent to which we can glue the paper onto the plate. We have also not considered the optical properties of the cardstock in this study.

From the scan data of this artifact, we establish a coordinate system using spheres A, B, and C (origin located at center of sphere A, center of sphere B on the X axis, and center of sphere C on the XY plane), and then evaluate the center of sphere D and the center of the contrast target in that coordinate system. Because a sphere target appears spherical from any view, we expect the coordinate system established using the spheres to not be influenced by the orientation of the artifact. This is the main idea behind our approach, which is verified by monitoring the center of sphere D as a function of different orientations. We then calculate the range (difference between the maximum and minimum) of center coordinates for the contrast target as the artifact is presented to the TLS in different orientations. If the range of the coordinates, especially along X and Y, is significantly larger than that for sphere D, we can then conclude that the contrast target suffers from orientation-dependent errors. The Z coordinate of the target is calculated by projecting the (X, Y) center onto a least squares best fit plane of the target and is robust in comparison to the X and Y coordinates. We, therefore, focus on the variation in the X and Y coordinates as a function of target orientation. Our approach does not require reference instruments or calibrated objects and can be easily applied even by users with limited budgets.

We performed measurements on our artifact using two TLSs—a short-range TLS (up to 25 m of range) that uses phase-shift technology and a long-range TLS (up to 1 km of range) that uses time of flight technology. For each TLS, we performed the following scans. With the artifact about 5 m away from the TLS, and the TLS resolution set at a certain value, we performed 10 scans at each of five nominal yaw angles: −40°, −20°, 0°, 20°, 40° (for a total of 50 scans), and at each of five nominal pitch angles: −40°, −20°, 0°, 20°, 40° (50 scans). To be clear, these are the angles between the normal vector to the plane of the contrast target and the line joining the TLS and the center of the contrast target. We then changed the resolution and repeated the scans. We then moved the artifact to about 10 m from the TLS and repeated the scans for both resolutions. A total of 400 scans per TLS were thus acquired. Regarding the choice of resolutions selected for the experiments, we chose one value at the lower end and one value at the higher end of the range of resolutions allowed for that TLS. For the long-range TLS, we choose 28 points-per-degree (ppd) as the lower resolution because it is a commonly used value in the forensic community. We choose 109 ppd as the higher resolution as a tradeoff between the time taken to execute the scan versus acquiring high-density data. It took about 2 min to execute the scans at this resolution. The TLS allows one additional higher resolution option, but it would have taken exceedingly long to perform the scans at that resolution. For the short-range TLS, we choose 30 ppd as the lower resolution to closely match the lower range of the long-range TLS resolution. We chose 90 ppd as the higher resolution as that is the highest possible resolution we could choose for this TLS.

**Figure 4:**
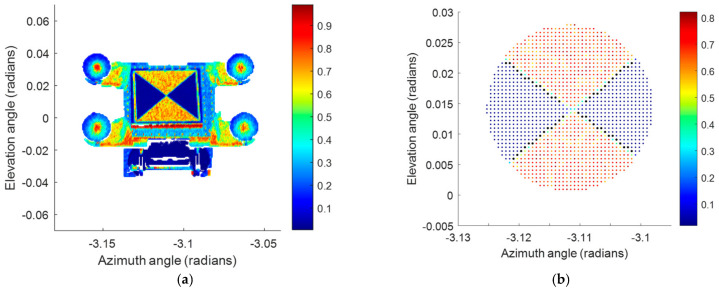
(**a**) Intensity plot of the entire artifact, (**b**) intensity plot of the contrast target and the edge points (transition between the black (blue dots in figure) and the white (red dots) regions of a target).

Each of the scan point clouds is first segmented into five parts—the four spheres, and the contrast target. The center coordinate from a least squares best fit sphere is found for each of the four sphere point clouds while the algorithm described in [42,43] is used to obtain the center of the contrast target. The sphere centers and contrast target centers are transformed into a coordinate system established using the centers of spheres A, B, and C. The center of sphere D and the center of the contrast target, in the previously established coordinate system, are then recorded for analysis. Figure 4a shows an intensity plot of the entire artifact while Figure 4b shows the intensity plot of the contrast target and the edge points (transition between the black (shown as blue dots in the figure) and the white (red dots) regions) used to find the center of the target.

Before we present the results, we note a few aspects of our technique regarding speed, accuracy, and cost. The time taken to perform a single scan of our artifact at a given rotation angle depends on the TLS, the resolution of the scan, and the distance of the artifact from the TLS. In our case, it took between thirty seconds and several minutes to complete a single scan. Performing 10 scans at each of the five angles took between 20 min to about 2 h. The entire set of measurements described in this paper took about two days to perform. The accuracy with which we can detect the change in the center is again dependent on the resolution of the TLS. As shown in the next section, we can detect center movement under a tenth of a millimeter for the TLSs we have considered. Finally, while the cost of building the artifact shown in Figure 2 might be significant for some users, there is an alternate lower-cost approach that might be suitable for most users. Four spheres and a contrast target can be mounted on a wall and the TLS could be moved to different positions to realize the different target orientations. Such an approach will be limited to the cost of acquiring four sphere targets, which is likely not prohibitively expensive.

## 4. Results

### 4.1. Visual Summaries of Bivariate Data Through Data Ellipses

Our discussion is focused on the X and Y coordinates since these are affected by the rotation of the target. The Z coordinate (the ranging direction of the TLS) is estimated by projecting the (X,Y) coordinate onto the plane of the target and is not impacted by target rotation.

A data ellipse provides a useful graphical summary of bivariate data (X and Y in our case). The centroid of a data ellipse represents the X and Y means of the data, its vertical and horizontal directions reflect the standard deviations and its tilt represents the correlation between the two variables. A data ellipse of radius *c* has a boundary given by $\varepsilon_{c}\left(\bar{y},\;S\right)=\bar{y}\;⨁\;cS^{1/2}$, where $\bar{y}=\left({\bar{y}}_{1},{\bar{y}}_{2}\right)$ is the mean vector of the two variables, S is the sample covariance matrix, and ⨁ indicates a scaling and rotation of the unit circle followed by a translation to the center $\bar{y}$ [49]. Often, the radius *c* is chosen as $c^{2}=qf(p,\;2,\;n-2)$, which is the *p*th percentile of an F-distribution with degrees of freedom 2 and n−2, with n indicating the sample size. This data ellipse will enclose approximately p∗100% of the data points when the two variables are bivariate normally distributed [49]. The choice of *p* may differ depending on the goal of the visualization. If the goal is to compare the similarity among groups, the primary interest is in assessing the degree of overlap in ellipses, in which case a large *p* corresponding to the majority of data points (e.g., 0.95) is appropriate. If instead, the focus is solely on comparing the noise (e.g., standard deviations) among groups, the choice of *p* is of less importance. In this case, the focus is on comparing the relative size of two (or more) ellipses, which will remain proportionally equivalent regardless of the scaling constant *c*, provided the same *c* is used for all groups. Data ellipses were generated in R version 4.3.2 [50] through the ggplot2 library [51].

In the following sections, we will use data ellipses to visualize the effect of distance, resolution, and pitch/yaw angle rotation on the X and Y coordinates of sphere D and the contrast target. In the visual summaries provided by the data ellipses, the focus of this paper is on comparing ellipse size and shifts in ellipse centers. We do not focus on any tilt in the ellipses, as the X and Y coordinates should be independent when the TLS is operating correctly (i.e., it is well compensated) and correlation estimates can be sensitive to outliers, particularly in small samples.

### 4.2. TLS I

We present results from the short-range TLS in this section. As mentioned in Section 3, with the artifact at a nominal distance (5 m or 10 m) away from the TLS, and the TLS set to a given resolution (30 ppd or 90 ppd), we performed 10 scans at each of five nominal yaw angles: −40°, −20°, 0°, 20°, 40° (for a total of 50 scans), and at each of five nominal pitch angles: −40°, −20°, 0°, 20°, 40° (50 scans). This process was repeated for every combination of distance and resolution, for a total of 400 scans. For each scan, we calculate the center of sphere D and the center of the contrast target in a coordinate system established using spheres A, B, and C.

**Figure 5:**
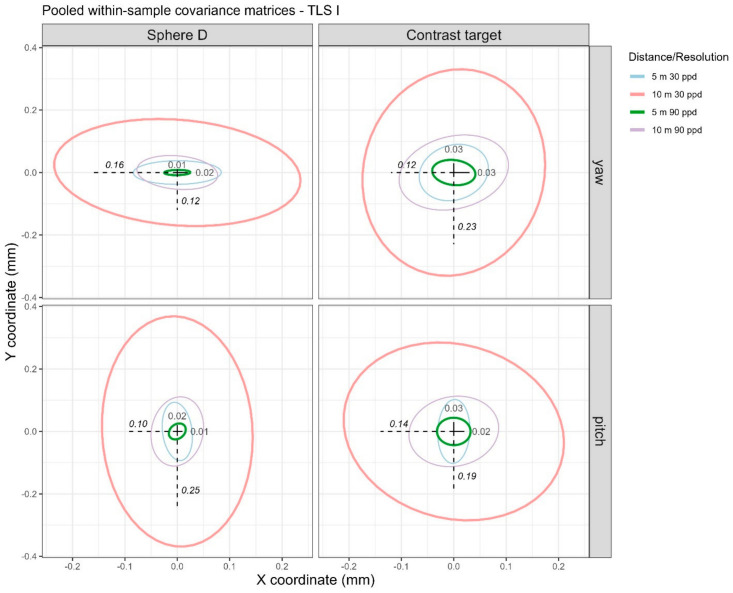
The 68% data ellipses visualizing the pooled within-sample covariance matrices for the four distance/resolution scenarios. Text annotations correspond to the standard deviations in the X (horizontal) and Y (vertical) coordinates for the far distance (10 m), low resolution (30 ppd) scenario (bolded and italicized values in Table 1), visualized by the magnitude of the dashed lines, and near distance (5 m), high resolution (90 ppd) scenario, indicated by solid lines (bolded values in Table 1).

We first address the noise levels (i.e., pooled one standard deviation) in the data before discussing systematic errors that may be present. For any given distance and resolution, we calculate the standard deviation of the coordinates from the 10 scans at each nominal angle value for each angle (pitch/yaw) and then calculate the pooled standard deviations across the five nominal angle values. Correspondingly, the pooled within-sample covariance matrices for the X and Y coordinates are visualized in Figure 5 through 68% data ellipses. The magnitude of the noise depends on both the distance of the artifact and the resolution of the scan, as expected. Noise levels are lowest, under 0.03 mm, at the near distance (5 m) and high resolution (90 ppd), illustrated in Figure 5 through the green (smallest) data ellipses. Noise levels are highest, between 0.10 mm and 0.25 mm, at the far distance (10 m) and low resolution (30 ppd), illustrated in Figure 5 by the red (largest) data ellipses. This is consistent across objects (sphere D or contrast target) and angle (for both pitch and yaw). While we do not show plots of the noise as a function of angle, they are reasonably constant across angle values, hence our decision to take the pooled standard deviations across the five nominal angle values.

**Table 1:** Pooled one standard deviation of the coordinates over the five yaw and five pitch angles for TLS I.

			Sphere D			Contrast Target		
Angle	Distance (m)	Resolution (ppd)	X (mm)	Y (mm)	Z (mm)	X (mm)	Y (mm)	Z (mm)
Yaw	5	30	0.06	0.03	0.02	0.05	0.06	0.01
	10	30	***0.16* ^†^**	***0.12***	0.07	***0.12***	***0.23***	0.04
	5	90	**0.02 ^‡^**	**0.01**	0.01	**0.03**	**0.03**	0.01
	10	90	0.05	0.04	0.03	0.07	0.08	0.02
Pitch	5	30	0.02	0.06	0.02	0.02	0.07	0.03
	10	30	***0.10***	***0.25***	0.08	***0.14***	***0.19***	0.08
	5	90	**0.01**	**0.02**	0.01	**0.02**	**0.03**	0.01
	10	90	0.03	0.08	0.03	0.06	0.08	0.03

^‡^ Bolded values have been added as text annotation to Figure 5, and they capture the lower end of the noise levels. ^†^ Bolded and italicized values capture the higher end of the noise levels.

**Figure 6:**
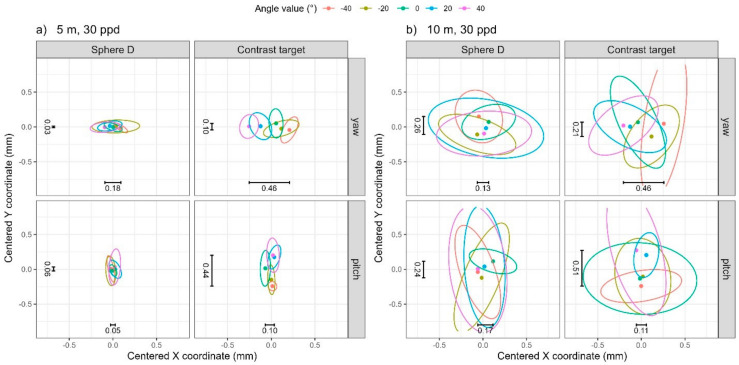
The 95% data ellipses from low-resolution scans (30 ppd) from TLS I for (**a**) 5 m distance and (**b**) 10 m distance. The range in the average X and Y coordinates from Table 2 have been added as text annotations.

**Figure 7:**
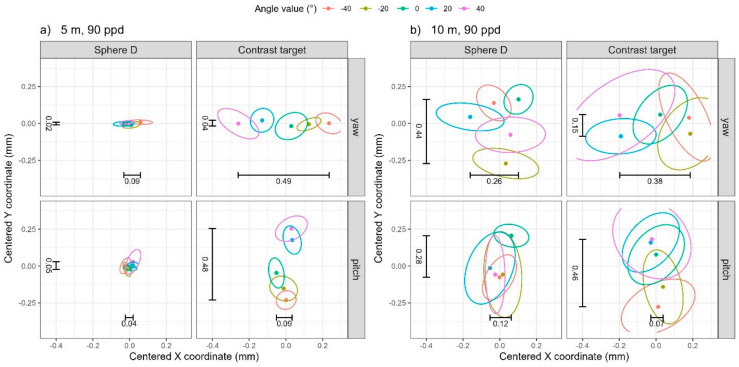
The 95% data ellipses from high-resolution scans (90 ppd) from TLS I for (**a**) 5 m distance and (**b**) 10 m distance. The range in the average X and Y coordinates from Table 2 have been added as text annotations.

Because we are interested in determining whether there are any systematic errors in the coordinate as a function of angle value, we calculate the mean coordinate from the 10 scans at each angle value (to attenuate the influence of noise). For any given distance and resolution, we report the range (difference between the maximum and minimum values) in the mean X, Y, and Z coordinates across the five angle values for each pitch and yaw in Table 2. The 95% data ellipses illustrating the (centered) data from the 10 scans at each angle value are shown in Figure 6 (low-resolution scans—30 ppd) and Figure 7 (high-resolution scans—90 ppd). Whereas Table 2 only contains the range in the mean coordinate, Figure 6 illustrates the shift in mean coordinates (ellipse centroids, represented as points) relative to the noise evident within each of the 10 scans (boundary of the ellipse).

For yaw rotation, the 5 m distance, and 30 ppd resolution (row 1 in Table 2), the range of the mean coordinate for the contrast target is 0.46 mm along X, which is significantly larger than that along Y (0.10 mm), and also larger than those for the sphere, indicating that the contrast target suffers from errors that depend on the yaw angle. This is illustrated in the top two panels of Figure 6a. The five data ellipses in the top right panel of Figure 6a (contrast target) demonstrate a significant shift in their average X coordinate relative to the ellipse boundaries, whereas no such shift is evident in the average Y coordinate, nor is any shift evident in the top left panel (Sphere D). It is interesting to note the trend in the mean X coordinate for the contrast target corresponds monotonically to an increase in yaw angle value. While we have not yet modeled and therefore cannot explain this trend, the impact of yaw rotations on the X coordinate is not surprising because the symmetry about the vertical axis, i.e., between the left and right side of the target, is affected by this rotation.

We observe similar behavior, but along the Y direction, for pitch rotation. At the 5 m distance and 30 ppd resolution (row 5 in Table 2), the range of the mean coordinate for the contrast target is 0.44 mm along Y, which is larger than that along X, and also larger than those for the sphere, indicating that the contrast target does suffer from errors that depend on the pitch angle. This is illustrated in the bottom two panels of Figure 6a. A trend is clearly visible in the Y coordinate for the contrast target and again, this is not surprising because the symmetry about the horizontal axis, i.e., between the top and bottom halves of the target, is affected by this rotation.

We observe similar trends in the X and Y coordinates at the 5 m distance and 90 ppd resolution (rows 3 and 6 in Table 2). While the range in the mean X and Y coordinates are comparable to those found at the 5 m and 30 ppd resolution, comparing Figure 7a to Figure 6a illustrates how the increased resolution decreases the noise (decreasing the overlap of the data ellipses) and accentuates the angle rotation effect. Note that the scale for Figure 6 is different than the scale used for Figure 7.

At 10 m, the noise generally masks any shift in target coordinates due to angle rotation. For example, at the 10 m distance and 30 ppd resolution, Figure 6b, the trend is somewhat visible for the X coordinate in the case of yaw rotation and the Y coordinate for pitch rotation, but the magnitude of the range of coordinates is generally dwarfed by the noise. The same is true for the 10 m distance and 90 ppd resolution, Figure 7b.

In summary, we see errors in the X and Y coordinates of the contrast target on the order of about 0.5 mm as the target is presented at different orientations to the TLS. At a near distance (5 m), this is larger than the noise levels and is therefore attributable to the effect of target orientation. There is a clear trend in the coordinates depending on the direction of the rotation.

### 4.3. TLS II

**Figure 8:**
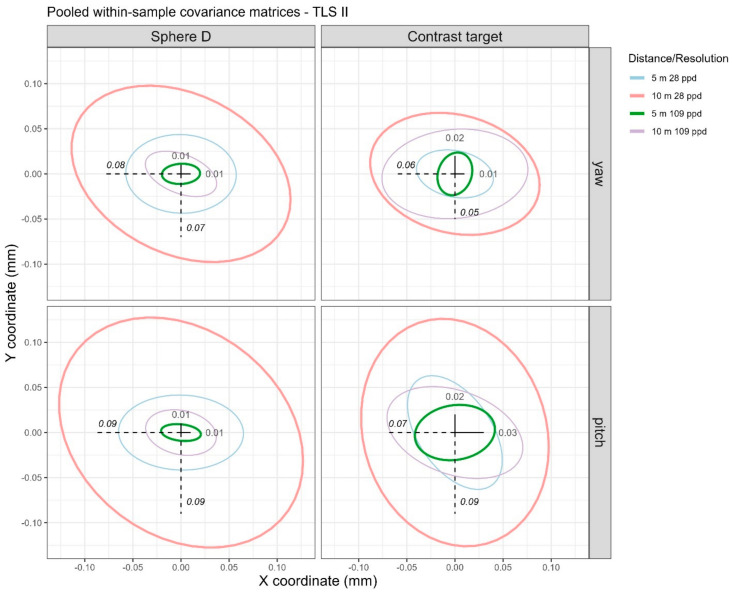
The 68% data ellipses visualizing the pooled within-sample covariance matrices for the four distance/resolution scenarios from the TLS II data. Text annotations correspond to the standard deviations in the X (horizontal) and Y (vertical) coordinates for the far distance (10 m), low resolution (28 ppd) scenario, visualized by the magnitude of the dashed lines (bolded and italicized values in Table 3), and near distance (5 m), high resolution (109 ppd) scenario, indicated by solid lines (bolded values in Table 3).

We present results from the long-range TLS in this section. The pooled one standard deviation values are shown in Table 3. As before, we first address the noise levels (i.e., pooled one standard deviation) in the data before discussing systematic errors that may be present. The magnitude of the noise depends on both the distance of the artifact and the resolution of the scan, as expected. Noise levels are lowest, under 0.03 mm, at the near distance (5 m) and high resolution (109 ppd) (rows 3 and 7 in Table 3) while they are highest, between 0.03 mm to 0.09 mm, at the far distance (10 m) and low resolution (28 ppd) (rows 2 and 6 in Table 3). This behavior is visualized through the pooled within-sample covariance matrices (X and Y coordinates) illustrated in Figure 8 through 68% data ellipses.

**Figure 9:**
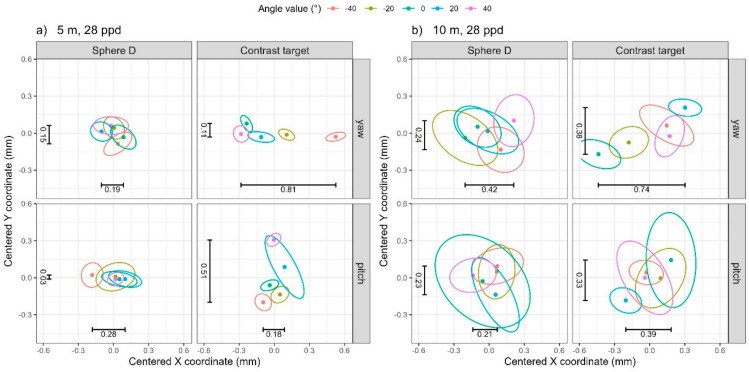
The 95% data ellipses from low-resolution scans (28 ppd) from TLS II for (**a**) 5 m distance and (**b**) 10 m distance. The range in the average X and Y coordinates from Table 4 have been added as text annotations.

The range of the mean coordinates from the 10 scans at each of the five yaw or five pitch angles is shown in Table 4. For yaw rotation, the 5 m distance, and 28 ppd resolution (row 1 in Table 4), the range of the mean coordinate for the contrast target is 0.81 mm along X, which is significantly larger than that along Y and also larger than those for the sphere, indicating that the contrast target suffers from errors that depend on the yaw angle. This is illustrated in the top two panels of Figure 9a, where the data ellipses in the top right panel (contrast target) demonstrate a significant shift in their average X coordinate relative to the ellipse boundaries, whereas no such shift is evident in the average Y coordinate, nor is any shift evident in the top left panel (Sphere D). The trend in the X coordinate is similar to that observed for TLS I.

We observe similar behavior, but along the Y direction, for pitch rotation. At the 5 m distance and 28 ppd resolution (row 5 in Table 4), the range of the mean coordinate for the contrast target is 0.51 mm along Y which is larger than that along X, and also larger than those for the sphere, indicating that the contrast target does suffer from errors that depend on the pitch angle. This is illustrated in the bottom two panels of Figure 9a. The trend in the Y coordinate is similar to that observed for TLS I.

**Figure 10:**
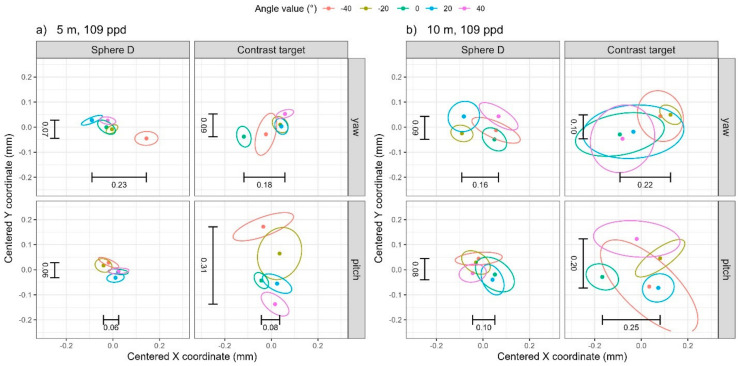
The 95% data ellipses from high-resolution scans (109 ppd) from TLS II for (**a**) 5 m distance and (**b**) 10 m distance. The range in the average X and Y coordinates from Table 4 have been added as text annotations.

For both yaw and pitch rotations, the 10 m distance and 28 ppd resolution (rows 2 and 6 in Table 4), see Figure 9b, the range of the mean coordinates for the contrast target are larger than those for the sphere, but the effect of yaw/pitch angle on the X and Y coordinates is masked by the noise in the low quality and quantity of data. At 109 ppd resolution, the range of coordinates for the contrast target is generally comparable with that of the sphere for both yaw and pitch rotations, see Figure 10. A notable exception is illustrated in the bottom panels of Figure 10a. Changing pitch rotation clearly affects the Y coordinate for the contrast target (bottom right panel) but has minimal effect on the Y coordinate for the sphere target (bottom left panel). Notably, the trend in the Y coordinate for the contrast target is reversed from that observed at the 5 m 28 ppd resolution (Figure 9a bottom right panel). We are not able to offer an explanation currently because we have not yet modeled this effect.

In summary, we see errors in the X and Y coordinates of the contrast target that depend on the resolution of the scan. At 28 ppd resolution, we see errors of about 0.3 mm to 0.8 mm as the target is presented at different orientations to the TLS. At 109 ppd resolution, the errors are under 0.3 mm and are generally comparable with that of the sphere.

## 5. Conclusions

Contrast targets are used in the scene to fuse data from different stations of the TLS or to fuse data from a TLS and other instruments. The nature in which a TLS captures data, the finite resolution of the TLS, and the planar geometry of the contrast target, all combine to introduce errors in the center of the target that depend on the orientation of the target with respect to the TLS. In this paper, we present a low-cost approach to quantify the magnitude of these errors. The work described in this paper is motivated by an ongoing documentary standards development activity within OSAC to devise a test procedure using contrast targets for TLS evaluation in the field by forensic practitioners.

To assess the orientation-dependent errors, we built an artifact comprising four spheres mounted on the corners of a large plate with the contrast target glued at the center. A coordinate system is established using three of the spheres and the center of the fourth sphere and the center of the contrast target are evaluated in this coordinate system. As the artifact is presented to the TLS in different orientations, the range of the coordinates of the center of the contrast target is a measure of the errors in the target as a function of angle. The fourth sphere serves as a control in that the range of errors in the center of the sphere is a measure of the overall errors due to the TLS and the measurement process.

Our experiments with two TLSs performed at two distances (5 m and 10 m) and two resolutions indicate that contrast target centers do in fact vary by a significant amount. For the short-range TLS, we see errors in the X and Y coordinates of the contrast target on the order of about 0.5 mm as the target is presented at different orientations to the TLS. There is also a clear trend in the X coordinate of the target as a function of yaw rotation and a trend in the Y coordinate as a function of pitch rotation. For the long-range TLS, we see errors in the X and Y coordinates of the contrast target that depend on the resolution of the scan. At 28 ppd resolution, we see errors of about 0.3 mm to 0.8 mm as the target is presented at different orientations to the TLS. At 109 ppd resolution, the errors are under 0.3 mm and comparable with that of the sphere.

While we built an artifact comprising four spheres and a contrast target mounted on a two-axis stage, the experiment can be realized easily by mounting four spheres and a contrast target on a wall and moving the TLS to different positions (horizontally and vertically) to realize the different orientations. Our approach offers greater control in conducting the experiment but targets on a wall might allow a user to assess orientation-dependent target errors in the field in a cost-effective and timely manner.

## Figures and Tables

**Table 2:** Range of mean coordinates for TLS I.

			Sphere D			Contrast Target		
Angle	Distance (m)	Resolution (ppd)	X (mm)	Y (mm)	Z (mm)	X (mm)	Y (mm)	Z (mm)
Yaw	5	30	0.18	0.03	0.02	0.46	0.10	0.07
	10	30	0.13	0.26	0.10	0.46	0.21	0.10
	5	90	0.09	0.02	0.00	0.49	0.04	0.12
	10	90	0.26	0.44	0.08	0.38	0.15	0.06
Pitch	5	30	0.05	0.06	0.04	0.10	0.44	0.18
	10	30	0.17	0.24	0.04	0.11	0.51	0.18
	5	90	0.04	0.05	0.04	0.09	0.48	0.15
	10	90	0.12	0.28	0.13	0.07	0.46	0.12

**Table 3:** Pooled one standard deviation of the coordinates over the five yaw and five pitch angles for TLS II.

			Sphere D			Contrast Target		
Angle	Distance (m)	Resolution (ppd)	X (mm)	Y (mm)	Z (mm)	X (mm)	Y (mm)	Z (mm)
Yaw	5	28	0.04	0.03	0.03	0.03	0.02	0.02
	10	28	***0.08* ^†^**	***0.07***	0.07	***0.06***	***0.05***	0.04
	5	109	**0.01 ^‡^**	**0.01**	0.01	**0.01**	**0.02**	0.02
	10	109	0.03	0.02	0.02	0.05	0.03	0.03
Pitch	5	28	0.04	0.03	0.03	0.03	0.04	0.03
	10	28	***0.09***	***0.09***	0.06	***0.07***	***0.09***	0.03
	5	109	**0.01**	**0.01**	0.01	**0.03**	**0.02**	0.03
	10	109	0.03	0.02	0.02	0.05	0.03	0.04

^‡^ Bolded values have been added as text annotation to Figure 8, and they capture the lower end of the noise levels. ^†^ Bolded and italicized values capture the higher end of the noise levels.

**Table 4:** Range of mean coordinates for TLS II.

			Sphere D			Contrast Target		
Angle	Distance (m)	Resolution (ppm)	X (mm)	Y (mm)	Z (mm)	X (mm)	Y (mm)	Z (mm)
Yaw	5	28	0.19	0.15	0.13	0.81	0.11	0.13
	10	28	0.42	0.24	0.25	0.74	0.38	0.21
	5	109	0.23	0.07	0.04	0.18	0.09	0.28
	10	109	0.16	0.09	0.13	0.22	0.10	0.19
Pitch	5	28	0.28	0.03	0.05	0.18	0.51	0.18
	10	28	0.21	0.23	0.24	0.39	0.33	0.47
	5	109	0.06	0.06	0.06	0.08	0.31	0.22
	10	109	0.10	0.08	0.07	0.25	0.20	0.18

## Data Availability

Data sharing is not applicable.
